# Stereochemically-aware bioactivity descriptors for uncharacterized chemical compounds

**DOI:** 10.1186/s13321-024-00867-4

**Published:** 2024-06-18

**Authors:** Arnau Comajuncosa-Creus, Aksel Lenes, Miguel Sánchez-Palomino, Dylan Dalton, Patrick Aloy

**Affiliations:** 1 Institute for Research in Biomedicine (IRB Barcelona), The Barcelona Institute of Science and Technology, Barcelona, Catalonia Spain; 2 Institució Catalana de Recerca i Estudis Avançats (ICREA), Barcelona, Catalonia Spain

**Keywords:** Small molecule bioactivity descriptors, Stereochemistry, Target-compound binding

## Abstract

Stereochemistry plays a fundamental role in pharmacology. Here, we systematically investigate the relationship between stereoisomerism and bioactivity on over 1 M compounds, finding that a very significant fraction (~ 40%) of spatial isomer pairs show, to some extent, distinct bioactivities. We then use the 3D representation of these molecules to train a collection of deep neural networks (*Signaturizers3D*) to generate bioactivity descriptors associated to small molecules, that capture their effects at increasing levels of biological complexity (i.e. from protein targets to clinical outcomes). Further, we assess the ability of the descriptors to distinguish between stereoisomers and to recapitulate their different target binding profiles. Overall, we show how these new stereochemically-aware descriptors provide an even more faithful description of complex small molecule bioactivity properties, capturing key differences in the activity of stereoisomers.

**Scientific contribution**

We systematically assess the relationship between stereoisomerism and bioactivity on a large scale, focusing on compound-target binding events, and use our findings to train novel deep learning models to generate stereochemically-aware bioactivity signatures for any compound of interest.

**Supplementary Information:**

The online version contains supplementary material available at 10.1186/s13321-024-00867-4.

## Main text

Small molecules are a great tool to probe biology and, still, the main asset of pharmaceutical companies. The last years have seen a surge of ever more complex biological high-throughput assays involving the use of chemical compounds, and databases committed to gathering bioactivity data associated to small molecules are expanding [1, 2]. Moreover, the widespread availability of computational resources [3] and artificial intelligence techniques has been pivotal to leverage such amounts of data [4].

From the computational perspective, small molecules are typically characterized by numerical descriptors encoding physicochemical or topological features [5]. Compounds can be further described using their biological activities (e.g. the targets they interact with), which represents a complementary strategy that extends the small molecule similarity principle beyond conventional chemical properties [6]. Unfortunately, experimental bioactivity data are sparse and only available for a limited set of well-characterized compounds. To overcome these coverage issues, we recently trained a collection of deep neural networks able to infer bioactivity signatures for any compound of interest (i.e. *Signaturizers*), even when little or no experimental information is available for them [7]. The Signaturizers are able to infer 25 different bioactivity types, from target profiles to cellular responses or clinical outcomes. Moreover, the vector-like format of the resulting bioactivity descriptors enables them to be readily used in day-to-day cheminformatics tasks. For instance, we showed their utility to navigate the chemical space in a biologically relevant manner, unveiling shared mechanisms of action in the absence of chemical similarity. Additionally, we demonstrated that small molecule bioactivity descriptors provide a significant improvement in performance, with respect to chemistry-restricted trained classifiers, across a series of biophysics and physiology activity prediction benchmarks. Indeed, our results showed that the added value of bioactivity descriptors increased together with the biological complexity of the classification tasks [7]. However, the original Signaturizers are built on 2D representations of molecules and are thus not able to capture subtle, but often meaningful, bioactivity differences between stereoisomers. Indeed, stereochemistry and chirality play pivotal roles in pharmacology [8, 9], often driving supramolecular recognition processes crucial in drug design. Biological matter is intrinsically chiral (e.g. amino acids) [10] and stereoisomeric small molecule drugs may exhibit different therapeutic and toxicological effects [11, 12]. For example, the antidepressant Citalopram is administered as a mixture of two enantiomers (i.e. racemate), although only one of them is active [13, 14]. However, in some other cases, one of the enantiomers is associated with toxic side effects. This is the case of the antiarthritic drug Penicillamine, administered as an enantiomerically pure compound ((*S*)-Penicillamine) since (*R*)-Penicillamine acts as a pyridoxine (vitamin B_6_) antagonist and is thus toxic [12, 15]. We now present novel deep learning models to generate stereochemically-aware bioactivity signatures for any compound of interest, which we call *Signaturizers3D*, that overcome the inherent limitations of our original Signaturizers.

### Systematic quantification of the relationship between stereochemistry and small molecule bioactivity

The first steps in the development of Signaturizers3D were (i) to select a comprehensive database containing detailed bioactivity data for a wide range of chemical compounds, and (ii) within this database, systematically identify groups of stereoisomers to compare their bioactivity profiles and evaluate the ability of Signaturizers3D to distinguish them.

**Fig. 1:**
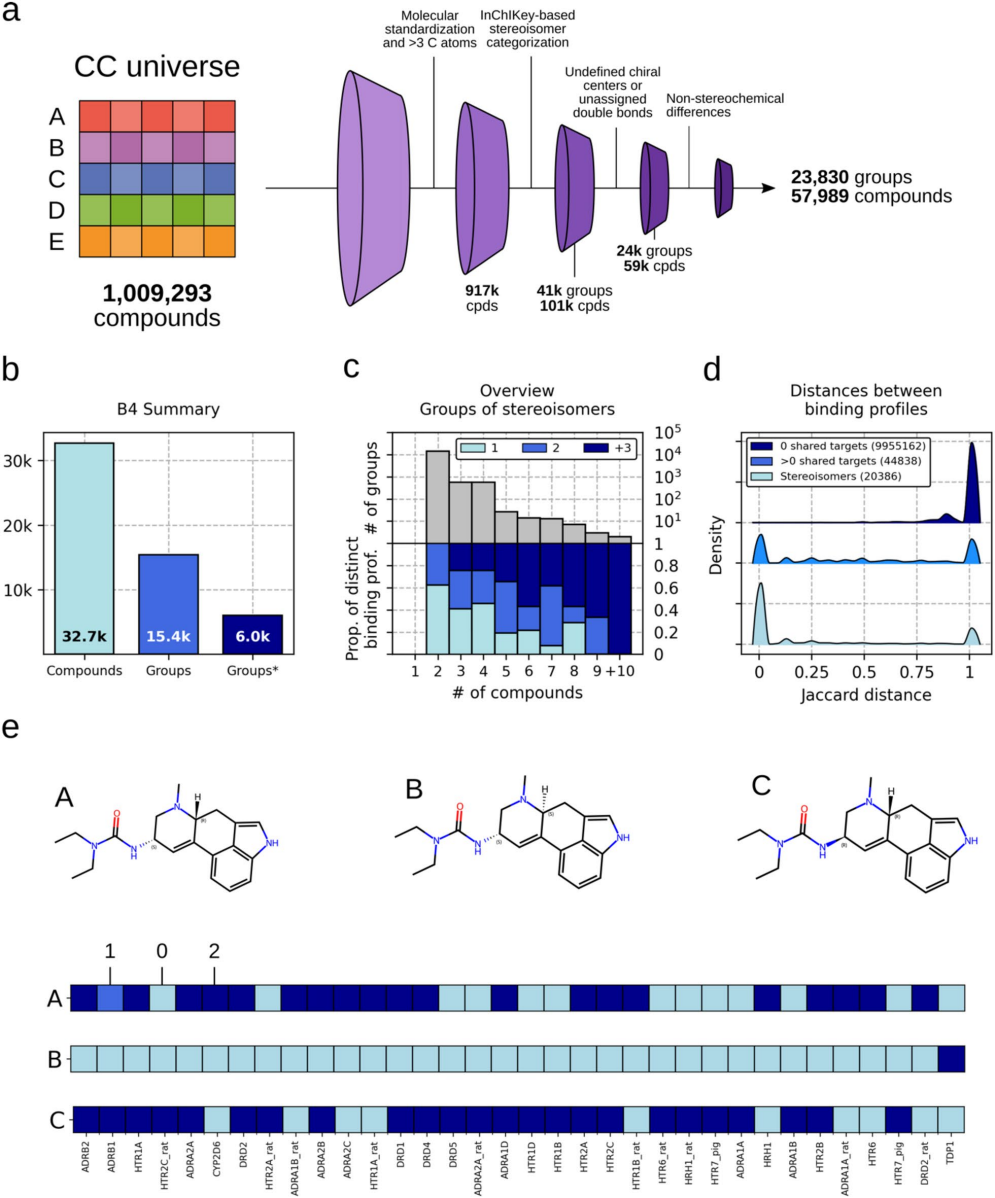
Stereoisomerism and bioactivity. **a** Computational pipeline to identify groups of stereoisomers in the CC chemical universe. **b** Number of unique stereoisomeric compounds with experimentally identified protein targets in the CC B4 space, number of stereoisomer groups, and number of groups with at least 2 compounds with non-identical binding profiles. **c** Number of groups (y-axis, top) having the specified number of stereoisomers (x-axis). Proportion of these groups (y-axis, bottom) having the specified number of distinct binding profiles (i.e. ~ 60% of the groups of 2 isomers have a unique binding profile). **d** Distributions of Jaccard distances (binding profiles) between pairs of compounds sharing 0, ≥ 1 targets and stereoisomer pairs. All distributions are significantly different from each other (Mann–Whitney p-value ~ 0). **e** Illustrative example of a stereoisomer group including 3 small molecules with their corresponding target binding profiles, using the annotation of type 0 signatures (i.e. 0: no binding; 1: weak binding and 2: strong binding)

To gather bioactivity data, we used the Chemical Checker (CC), which represents the largest collection of small molecule bioactivity signatures available to date, with experimental information for over 1 M compounds [6]. The CC divides data into five levels of increasing complexity, ranging from the chemical properties of compounds to their clinical outcomes. Compound bioactivities are expressed in a vector-like format (i.e. signatures), and the data processing pipeline also includes several steps of increasing level of integration and abstraction: from raw experimental data representing explicit knowledge (type 0 signatures) to inferred representations that leverage all the experimentally determined bioactivities available for each molecule (type III signatures). Thus, we processed the whole CC (i.e. 25 different bioactivity types for about 1 M molecules) to systematically identify groups of stereoisomers that might exhibit distinct bioactivities. In brief, we first identified stereoisomers using their InChIKey strings and we then applied several filters to ensure that the actual differences between compounds were exclusively due to stereochemical variations (see Supplementary Information for further details). Then, we selectively removed molecules that were not exhaustively characterized, in order to work with enantiomerically pure compounds and prevent the analysis of results derived from racemic mixtures (Fig. 1a). We eventually identified 23,830 groups of stereoisomers, involving 57,989 compounds, across the different CC bioactivity spaces. We found most stereoisomeric groups with experimental information in the target binding space (B4) and in the network spaces derived from B4 (i.e. C3–5, Fig S1). We thus focused our study on the B4 space, which contains over 600,000 molecules, and we identified 15,370 groups of stereoisomers, involving 32,705 compounds (Fig. 1b). We then analyzed the binding profiles for all these compounds, and found 6022 groups that had at least 2 stereoisomers with non-identical binding profiles. We also observed that the majority of the groups (14,181, ~ 92%) contained only 2 stereoisomers (Fig. 1c, top), in 38% of which both compounds showed distinct binding profiles (Fig. 1c, bottom). Analogously, we identified 562 groups containing 3 stereoisomers: 230 (41%), 195 (35%) and 137 (24%) of them showing 1, 2 and 3 distinct binding profiles, respectively. Finally, we observed that the distribution of Jaccard distances between binding profiles within stereoisomeric groups was skewed towards low values (i.e. more similar profiles) compared with random pairs, while pairs of compounds sharing at least one target were somewhere in the middle (Fig. 1d). Figure 1e shows, as an illustrative example, a group of 3 stereoisomers with non-identical binding profiles, where compounds A and C weakly and strongly bind with the Beta-1 adrenergic receptor (ADRB1; 2nd position in the profile), respectively, whilst compound B does not bind it. Note that inactive compound-target interactions might be false negatives due to, for instance, a limited sensitivity of the detection methods or non-tested enantiomers.

### Design and evaluation of stereochemically-aware Signaturizers

**Fig. 2:**
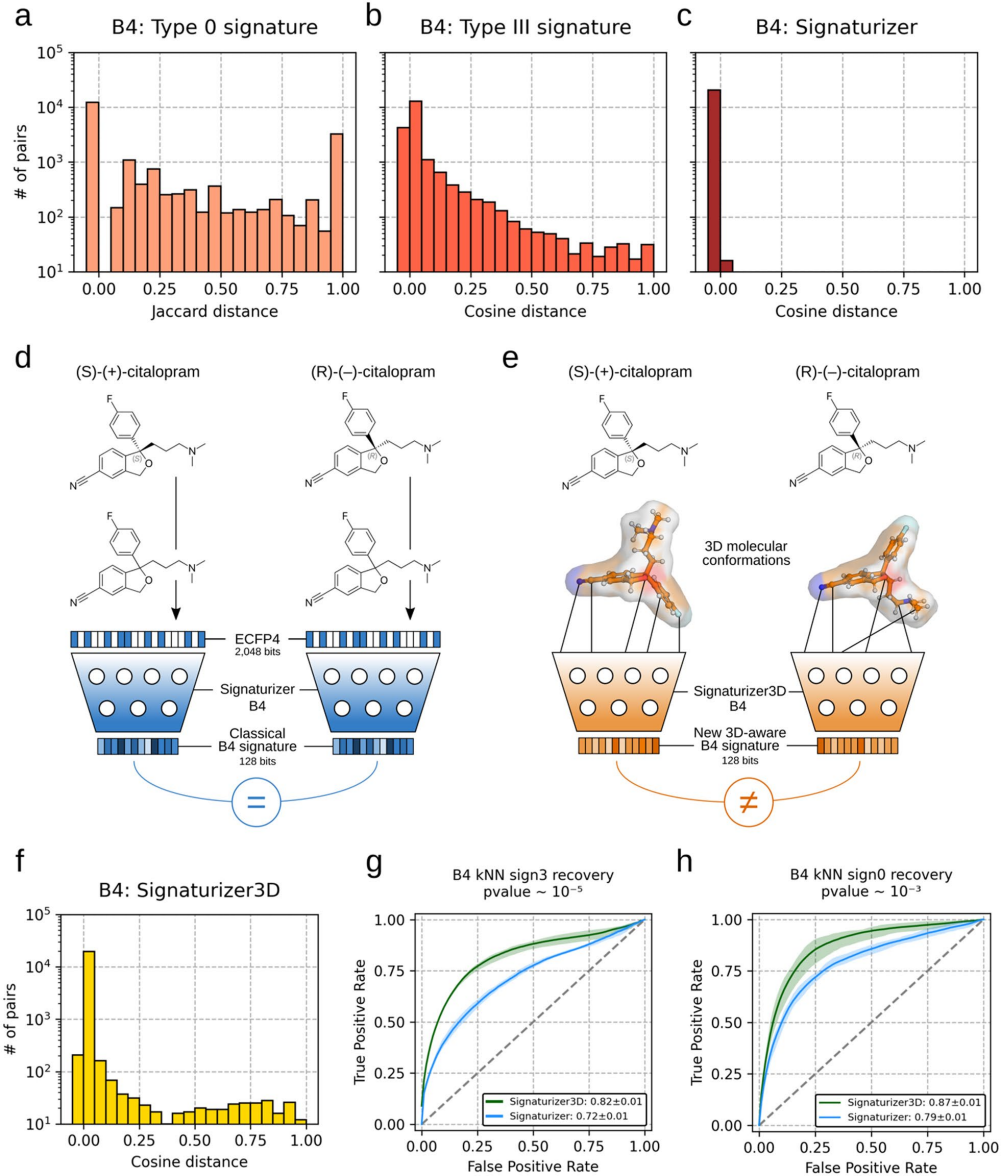
Stereochemically-aware bioactivity descriptors. **a** Distribution of target binding profile Jaccard distances (CC B4 type 0 signatures) between stereoisomer pairs (20,386 pairs). **b** Distribution of CC B4 type III signature cosine distances between stereoisomer pairs. **c** Distribution of Signaturizer cosine distances between stereoisomer pairs. **d** Graphical scheme of the *signaturization* process of distinct stereoisomers ((*S*)-(+)-citalopram and (*R*)-(−)-citalopram) with the Signaturizer. Molecules are first represented by 2D-based fingerprints (ECFP4, 3D information is lost) and then input to a neural network. Since ECFP4 for both stereoisomers are identical, output signatures are also identical. **e** Graphical scheme of the *signaturization* process of distinct stereoisomers ((*S*)-(+)-citalopram and (*R*)-(−)-citalopram) with the novel Signaturizer3D. 3D conformations are first generated for both molecules and the corresponding molecular representations are input to the Signaturizer3D fine-tuned neural network. Since molecular representations for both stereoisomers are different, output signatures are also different. **f** Distribution of Signaturizer3D cosine distances between stereoisomer pairs. **g** Recapitulation of B4 signature type III kNNs (×3 80/20 splits) using the original Signaturizer and the Signaturizer3D. Nearest neighbors are defined as those molecules with a B4 cosine distance to the evaluated compound in the 0.001 percentile of the distribution (p-value ~ 10^–5^). **h** Recapitulation of B4 signature type 0 kNNs using the original Signaturizer and the Signaturizer3D. Nearest neighbors are defined as those molecules with a B4 cosine distance to the evaluated compound in the 0.1 percentile of the distribution (p-value ~ 10^–3^). To speed up the comparisons, positive (NN) and negative (non-NN) pairs were subsampled (10 × 2.5 k compounds) from the CC B4 space

Our analyses showed that most stereoisomer pairs (60.4%) had identical target profiles but, perhaps more interestingly, the remaining 8081 pairs (39.6%) showed distinct binding against protein targets (Fig. 2a). We also observed that CC type III signatures captured differences between stereoisomer pairs (Fig. 2b). However, these differences were fully missed by the Signaturizers (Fig. 2c), as they were trained on 2D representations of the chemical molecules (i.e. ECFP4 [16]), highlighting the need to develop new descriptors able to distinguish stereoisomer-specific bioactivities.

To overcome the limitations of the original Signaturizers, we trained new deep-networks using 3D-aware molecular representations (i.e. Signaturizers3D, Fig. 2e). We first generated 3D conformations for all CC molecules, coupled them with their type III signatures, and used them to fine-tune the pre-trained Uni-Mol model [17]. In brief, for all molecules in the CC, we generated and optimized a single 3D conformation per compound using the ETKDG method [18] and the Merck Molecular Force Field (MMFF94) from RDKit. After removing hydrogens, all coordinates and atom-types for each molecule were used to fine-tune the pre-trained Uni-Mol model as a multitarget regression problem, so that we could directly infer pre-calculated CC type III signatures (128 dimensions). Specific details regarding the training of the models are provided in the Supplementary Information. We then evaluated the capability of Signaturizers3D to distinguish stereoisomers by generating B4 signatures for the 32,705 compounds identified as stereoisomers in the CC B4 space and calculating distances between them. We found that, opposed to the original Signaturizers, virtually all pairs of stereoisomers (99.9%) exhibited non-identical 3D signatures (Fig. 2f), showcasing the ability of our new models to capture slight differences in the stereochemistry of the compounds. Next, we followed a strict approach to assess the ability of Signaturizers3D to recapitulate k-nearest neighbor (kNN) compounds at type III signature level; this is to evaluate their capacity to retain the structure of the original data similarity. In brief, in a standard kNN recovery task, negative pairs are chosen randomly and can differ significantly from positive pairs (Fig S2a). We used the same strategy to evaluate the capacity of the new descriptors to retain traceable biological information (e.g. type 0 signatures), in the form of compound-target pairs (Fig S2b). Under this scenario, both Signaturizers and Signaturizers3D could almost perfectly distinguish close from distant molecules at type III and 0 signatures level. To make the assessment more stringent and realistic, we selected the negatives within a close distance of the molecule under evaluation, making the discernment between positive and negative pairs a more difficult task (Fig S2c; see Supplementary Information). In this case, we observed that, indeed, Signaturizers3D were able to better recapitulate type III and 0 signatures than the original ECFP4-based Signaturizers (Fig. 2g, h).

## Conclusions

We have systematically assessed the relationship between stereoisomerism and bioactivity on a large scale, focusing on compound-target binding events. Subsequently, we used our findings to train the second generation of Signaturizers, which are now stereochemically-aware, thereby providing an even more faithful and accurate representation of complex small molecule bioactivity properties.

### The Signaturizer3D package

An open source Python package to generate 3D-aware CC bioactivity signatures is available at https://gitlabsbnb.irbbarcelona.org/packages/Signaturizer3d. The package includes model weights for each of the 25 CC spaces and can be used to characterize molecules using SMILES or coordinates from existing conformers as input. The models are implemented in Pytorch and support inference on a GPU or CPU. The average time to generate CC signatures from SMILES is 16.3 s per 1000 molecules on an NVIDIA GeForce RTX 3090.

### Supplementary Information

Supplementary Material 1.

## Data Availability

No datasets were generated or analysed during the current study.
