# Supplementary material for: Stereochemically-aware bioactivity descriptors for uncharacterized chemical compounds
Source: J Cheminform. 2024 Jun 18;16:70. doi: 10.1186/s13321-024-00867-4 (PMC11186078; doi:10.1186/s13321-024-00867-4)
Supplement: Supplementary file 1 — Supplementary Material 1. [file 13321_2024_867_MOESM1_ESM.docx]

**Supplementary Information**

*Identification of stereoisomers in the CC universe*

We first downloaded small molecule bioactivity data from the official CC website (https://chemicalchecker.com/downloads/signature0, 25 spaces). We standardized a total number of 1,009,293 compounds (InChIs) using the *standardiser* package (https://github.com/flatkinson/standardiser/tree/master, removal of solvent and salt molecules, charge neutralization and application of tautomeric rules), leading to 916,931 standardized and neutral small molecules having 3 or more carbon atoms. Subsequently, we identified groups of stereoisomers based on their InChIKeys [19], generated using RDKit (https://www.rdkit.org). For compounds to be categorized in the same group, they needed to have identical first blocks (indicating identical connectivities between atoms) and non-identical second blocks (indicating differences in stereochemistry, isotopic atoms, the InChI version, etc). In addition, we excluded small molecules with unspecified chiral centers or unassigned double bond configurations. Finally, we also removed compound pairs having different InChIs after stereochemical information was deleted using RDKit, such as pairs with distinct isotopes. We only conserved groups having ≥2 compounds. Overall, we identified 23,830 groups of stereoisomers, including 57,989 compounds. A graphical scheme of the pipeline is shown in **Fig 1a**.

*Training and evaluation of Signaturizers3D*

For all molecules in the CC, we generated and optimized a single 3D conformation per compound using the ETKDG method [18] and the Merck Molecular Force Field (MMFF94) from RDKit, respectively, with hydrogens included. We successfully generated conformations for ~99% of the compounds (998,186). The rest, mostly very large molecules, were excluded from the final dataset. We then did 80/20 scaffold-based splits (x3) of the 998,186 considered compounds.

After removing hydrogens, all coordinates and atom-types for each molecule were used to fine-tune the pre-trained Uni-Mol model as a multitarget regression problem, so that we could directly infer pre-calculated CC type III signatures (128 dimensions). Uni-Mol pre-trained model was directly downloaded from https://github.com/dptech-corp/Uni-Mol/releases/download/v0.1/mol_pre_no_h_220816.pt, which provided the initial weights and defined the model’s architecture. To fine-tune the existing Uni-Mol model, we trained for 40 epochs with a batch size of 32 and a learning rate of 0.0001 (warmup ratio of 0.06 without dropout). We used the Adam optimizer with betas (0.9, 0.99) and epsilon 10^-6^, and we utilized a polynomial decay scheduler for the learning rate. Models were trained on the previously generated scaffold-based splits (3x25, 80/20) using a smooth mean absolute error loss for function optimization. Validation was based on the aggregated mean absolute error (*valid_agg_mae*), with early stopping after 20 epochs if no improvement was observed. Training was executed on a GPU with mixed precision (FP16).

*K-nearest neighbors recovery*

First, we needed to establish a distance cutoff to eventually define positive pairs of compounds (nearest neighbors) at type III signature level. To do so, we sampled 10k molecules in each of the 25 bioactivity spaces and, after calculating the full pairwise distance matrix between these compounds (10^8^ distances within each space), we established the NN cutoff distance as the 0.001 percentile of the distribution. In this way, a distance cut-off was defined for each CC space.

Positive pairs (NN) for a given compound at type III signature level were defined as those compounds being at a distance lower than the established cutoff for each CC space. For the strict NN recovery, negative pairs (not NN) were defined as those being at a distance greater than the established cutoff but lower than three times the cutoff distance. The ratio of negative to positive pairs was capped at 10:1. We calculated distances between compounds using Signaturizers and Signaturizers3D for all (x3) scaffold-based 80/20 splits, and we assessed their ability to recapitulate NNs at type III signature level by calculating the corresponding ROC Curves with a subsample of 1k molecules per space. Analogously, positive pairs (NN) for a given compound at B4 type 0 signature level were defined as those compounds having identical target binding profiles (corresponding to a p-value ~10^-3^). For the strict NN recovery, negative pairs (not NN) were defined as those having a Jaccard distance <0.6 (any pair with a Jaccard distance >0 was accepted as negative in the classical NN recovery exercise). We calculated distances between compounds using Signaturizers and Signaturizers3D and we assessed their ability to recapitulate NNs at type 0 signature level by calculating the corresponding ROC Curves with x10 subsamples of 2.5k molecules.

**
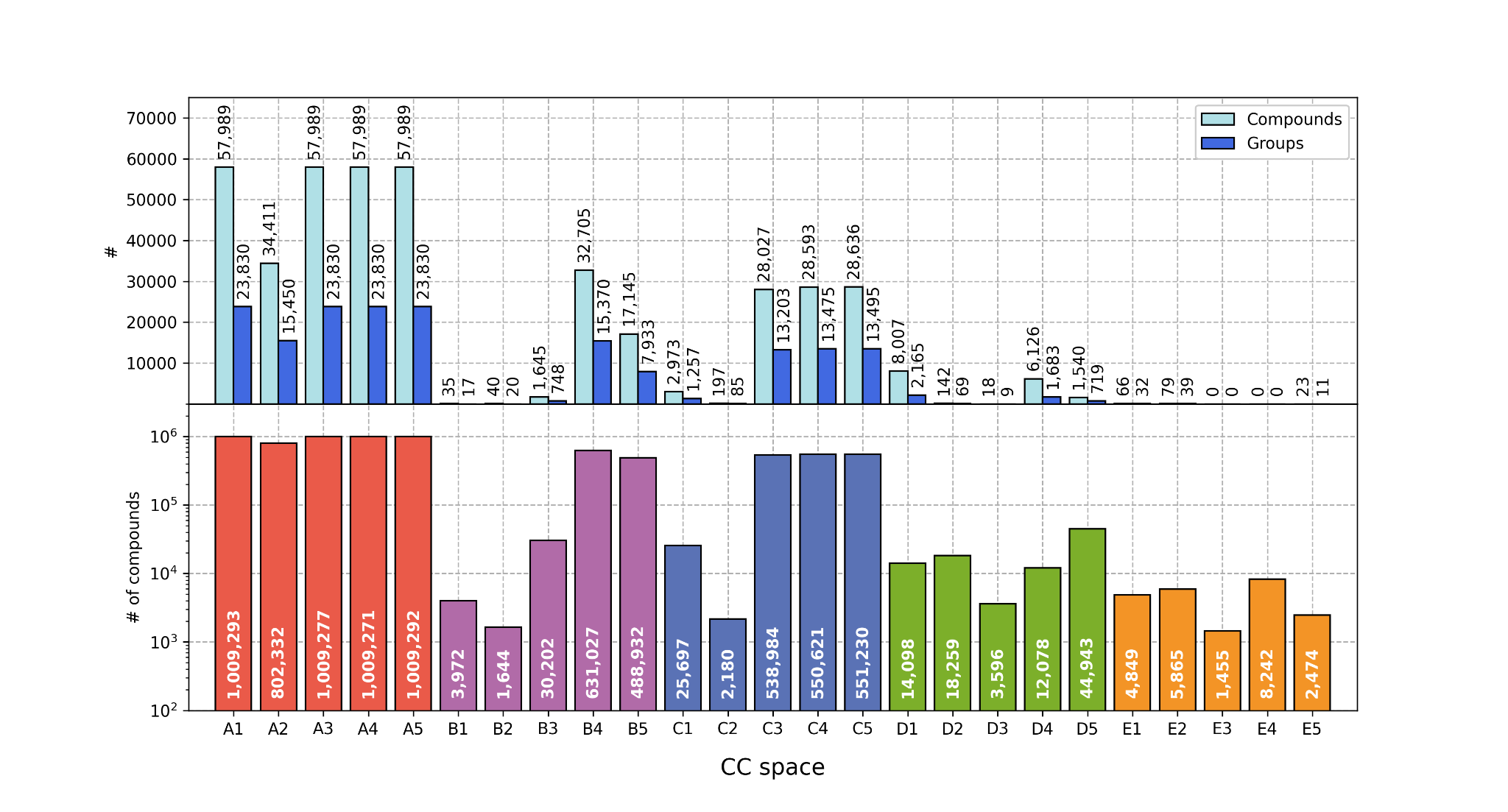
**

**Figure S1**: Number of compounds (bottom) in each CC space. Number of groups of stereoisomers and number of stereoisomers (top) in each CC space.

**
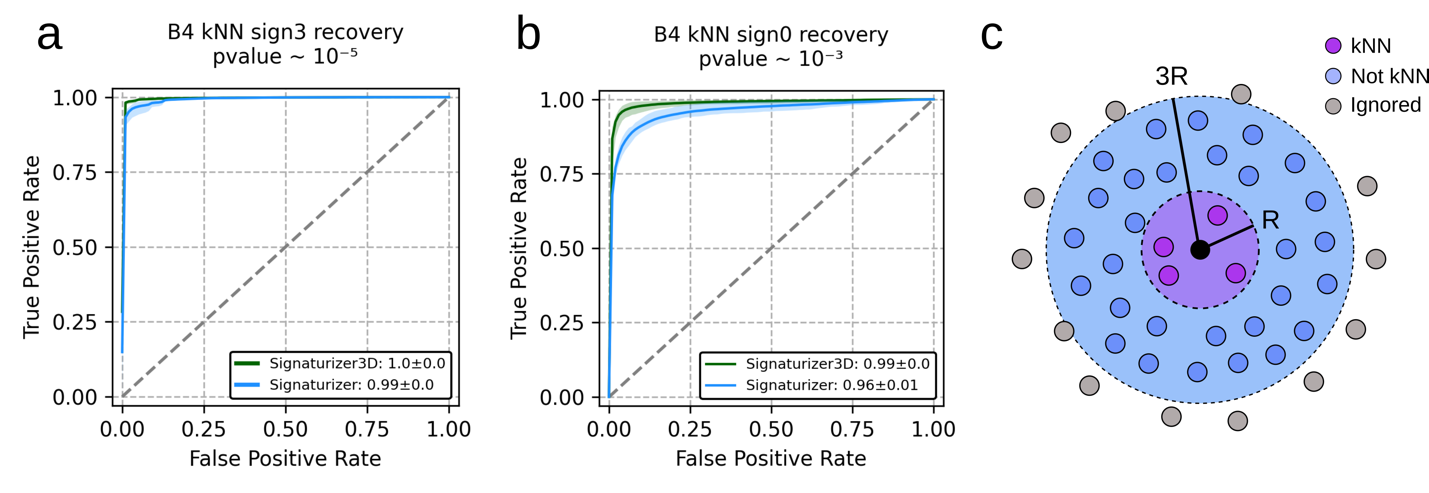
**

**Figure S2**: **a)** Recapitulation of B4 signature type III kNNs (x3 80/20 splits) using the original Signaturizer and the Signaturizer3D. Nearest neighbors were defined as those molecules with a B4 cosine distance to the evaluated compound in the 0.001 percentile of the distribution (p-value ~10^-5^). **b)** Recapitulation of B4 signature type 0 kNNs using the original Signaturizer and the Signaturizer3D. Nearest neighbors were defined as those molecules with a B4 cosine distance to the evaluated compound in the 0.1 percentile of the distribution (p-value ~10^-3^). **c)** Stringent strategy to assess the recovery of k nearest neighbors (kNN): given a seed compound (black), NNs are defined as those k compounds being at a distance <R (purple). On the other hand, non-NN are defined as those compounds being at a distance >R but <3R (blue), so that non-NN are still close to the real neighbors. All compounds at a distance >3R (gray) are ignored. The R was set to the 0.001 (pvalue ~10^-5^) and 0.1 (pvalue ~10^-3^) percentile of closest compounds for type III and 0 signatures, respectively, which reflect the different number of compounds and possible comparisons in each space.
